# Assessment of Selected Heavy Metals and Arsenic Concentrations in Wild Boar (*Sus scrofa* L.) from Papuk Nature Park (Croatia)

**DOI:** 10.3390/jox15030074

**Published:** 2025-05-15

**Authors:** Domagoj Vidosavljević, Miroslav Venus, Dinko Puntarić, Lidija Kalinić, Marina Vidosavljević, Mario Begović, Marta Despot, Vlatka Gvozdić

**Affiliations:** 1 Faculty of Medicine Osijek, Josip Juraj Strossmayer University of Osijek, J. Huttlera 4, HR 31000 Osijek, Croatia; domagoj.vidosavljevic@gmail.com; 2 Faculty of Dental Medicine and Health, Josip Juraj Strossmayer University of Osijek, Crkvena 21, HR 31000 Osijek, Croatia; venusmiroslav@gmail.com; 3 Faculty of Medicine, Croatian Catholic University of Zagreb, Ilica 244, HR 10000 Zagreb, Croatia; dinko.puntaric@unicath.hr; 4 Department of Biology, Josip Juraj Strossmayer University of Osijek, Cara Hadrijana 10, HR 31000 Osijek, Croatia; lkalinic@biologija.unios.hr; 5 Interdisciplinary Postgraduate Study in Molecular Biosciences, Josip Juraj Strossmayer University of Osijek, Cara Hadrijana 10 e, HR 31000 Osijek, Croatia; vidosavljevic.marina@gmail.com; 6 National Memorial Hospital Vukovar, Bolnicka 5, HR 32000 Vukovar, Croatia; begovicvu@gmail.com; 7 Department of Chemistry, Josip Juraj Strossmayer University of Osijek, Cara Hadrijana 8/A, HR 31000 Osijek, Croatia; mdespot@unios.hr

**Keywords:** heavy metals, arsenic, wild boar, Papuk Nature Park, chemometrics

## Abstract

The aim of this study was to measure the concentrations of As, Cd, Hg, Pb, Cr, Cu, Fe and Zn in the muscle, liver and kidney of wild boar (*Sus scrofa*) in Papuk Nature Park, Eastern Croatia. Muscles, liver and kidney of 38 wild boars, up to 3 years of age, were collected and the concentrations of elements were determined by ICP-MS. Cadmium exceeded the permitted levels acceptable for human consumption in 50% of kidney samples (max. = 6.64 mg kg^−1^), 20% of liver samples (max. = 4.60 mg kg^−1^) and 43% of muscle samples (max. = 0.672 mg kg^−1^). Lead exceeded acceptable levels in 63% of liver samples (max. = 0.463 mg kg^−1^), 51% of kidney samples (max. = 11.8 mg kg^−1^) and 65% of muscle samples (max. = 9.10 mg kg^−1^). Mercury concentrations in the liver were higher than allowed in 13% (max. = 0.552 mg kg^−1^) in kidneys in 27% (max. = 0.484 mg kg^−1^), and in the muscles in 15% (max. = 0.103 mg kg^−1^) of cases. Arsenic concentrations in muscles exceeded the permissible value in 30% of cases (max. = 0.286 mg kg^−1^). The concentrations of Cr, Cu, Fe and Zn did not significantly differ from the values reported in other studies. Median concentrations of Cr, Cu, Fe and Zn ranged as follows: muscle (0.193, 2.94, 44.5 and 20.6 mg kg^−1^), kidney (0.140, 5.32, 110 and 23.3 mg kg^−1^) and liver (0.130, 3.71, 278 and 36.0 mg kg^−1^).

## 1. Introduction

**Figure 1:**
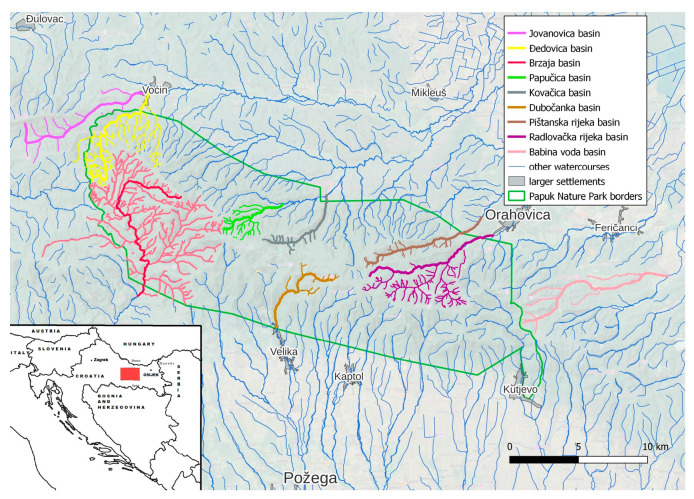
Map of the sampling area.

Papuk mountain represents a geological remnant of the Pannonian Sea situated in the vast Slavonian plain in the eastern part of Croatia. The region itself is situated on the border of Croatia and Hungary, separated by the river Drava (Figure 1). Due to its exceptional geological and biological diversity and valuable cultural heritage, Papuk became a member of the European and World Geoparks Network and was included in the UNESCO list of Geoparks as the first Croatian geopark. Papuk has a great forest and, as such, it is an excellent habitat for many animal species, and it is a refuge for deer, roe deer, wild boar, marten foxes and birds [1,2]. Within the Papuk Nature Park many areas differ in the degree of protection from other parts, and due to their unusual features, they received the status of specially protected areas. In addition, Papuk was a location of significant war activities during Homeland war (1991–1995).Villages were destroyed, and fields and forests contaminated with mine fields, which additionally contributed to metal contamination [3].

Wild boar (*Sus scrofa* L.) is an autochthonous large game species in Croatia and, similar to populations in all of Europe, is exhibiting growth. With more than 50,000 active members (about 1.15% of the Croatian population), hunters in Croatia represent a numerous and relatively influential social group connected by a similar way of behaving in their free time. Although to a much lesser extent than the meat of domestic animals, wild boar meat has always been part of the diet of the Croatian population. It is a well-known fact that domestic animals live in “controlled” conditions with regard to accommodation and movement, as well as their diets, while wild animals feed in a large area, have freedom of choice depending on seasonal availability and live, as a rule, longer than domestic animals.

When assessing potential biological hazards, one should consider the fact that wild boar meat in these areas is often added to traditional meat products—such as fermented sausages that are not subjected to any heat treatment—therefore representing a significant risk for consumers of game meat [4]. Toxic Cd, Hg and Pb levels in game meat may be relatively unimportant in terms of dietary intake of the Croatian population in general, but may have a significant impact when consumed on a regular basis, especially considering the background factors which influence Cd uptake and bioavailability (sex, age, living environment, smoking habits, etc.). Many elements are classified as heavy metals (Cd, Pb, Hg, Mn, Fe, Cu, Zn), but some are relevant in the context of environmental pollution. Essential heavy metals (Cr, Cu, Fe, Zn) are important for living organisms and may be needed by the body in low concentrations. Non-essential heavy metals (As, Cd, Pb, Hg) are toxic, have no known biological role in living organisms, and are considered biologically irrelevant [5].

A study among the hunters in Italy confirmed their more frequent consumption of game meat and offal (liver) than the general population. Consumption of wild animal intestines (livers) significantly contributes to increased exposure to cadmium and lead in members of hunter households, and, in principle, the consumption of offal should be kept to a minimum, especially for children from hunting households. However, some of these metals are also introduced to animals’ meat by bullets used during hunting. Wild boar might be the most suitable indicator of environmental pollution, because they are exposed to different contaminants by water and soil due to innate habits of omnivorous scavengers and soil ingestion due to digging during foraging [6].

The heavy metals and metalloids concentrations in domestic and wild animals differ greatly, sometimes regardless of age, and are higher in wild animals, with regard to what is found in kidneys [7]. In some studies, cadmium values were higher in older animals, and established mercury and lead values do not show a significant dependence on animal age while, in others, differences in lead, cadmium and mercury concentrations are found in relation to animal age [8].

The absence of continuous monitoring of the health status of game (the monitoring system applies only to domestic pigs) represents an additional problem in the analysis of risk assessments relevant to the use of meat in the diet. Metals in the environment represent a special problem because of their long lasting and direct effect on human health. Among all, As, Cd, Hg and Pb have been recognised as a major problem because of their unquestionable toxicity, excretion and accumulation in vital organs of animals and humans [9]. In addition to the environment, arsenic can also be found on bullets and can be transferred to the meat during hunting [10]. Inorganic mercury is a ubiquitous, persistent, bio-accumulative, toxic pollutant. In the environment, it can be introduced by anthropogenic activities such as mining, pesticides, the paper industry, etc. Cadmium absorbed from the soil and translocated in the plants can enter the food supply, thereby presenting a food safety concern. Other major sources include stabilisers for plastics, an alloying element of some lead, copper and tin alloys, batteries, fertilisers, weathering of rocks and other anthropogenic activities [11]. The greatest risk comes from the use of bullets made from lead, as these bullets tend to fragment and disperse across harvested game animals [12]. Once ingested, heavy metals and metalloids, in the long run, tend to affect major organs in the animal’s body, such as offal, skin, muscles, thyroid gland, lungs, etc.

During routine control in 2006 and 2007, unexpectedly high concentrations of heavy metals were found in game meat from the Papuk Nature Park. The determined concentrations of Cd and Pb were higher—or many times higher—than those prescribed for meat of farmed animals intended for human consumption. Elevated values have been established not only in Virovitica-Podravina County, where part of the Papuk Nature Park geographically belongs, but also in other counties in north-eastern Croatia [13,14,15].

Also, in a study conducted in Baranja in 2005, the detected values of metals in samples of deer kidneys were far above what is allowable, especially in older animals [13]. Apart from the certain presence of uranium that was found in the eastern part of Papuk, and which is considered to be of epigenetic origin, nothing indicates the possible presence of any metal contamination (and any other contaminants), since there were almost never permanently settled people in that area, and there has never been industry or heavy traffic [16].

In 2008, the Croatian regulation on maximum allowable levels of metals and metalloids was harmonised with EU legislation and, since then, the monitoring of Hg and As in animal tissues (other than fish) for human consumption was no longer obligatory [17]. Data about metal concentrations in game in Croatia are mainly limited to scientific research [18,19,20,21].

To our knowledge, this is the first study that systematically describes the concentrations of heavy metals (Cd, Hg, Pb) and metalloids (As) in the kidney, liver, and muscles of wild boars from the area of national park Papuk in eastern Croatia. Systematic research on the load of wildlife in the Papuk Nature Park has never been performed, so this paper covers all possible sources of metal contamination, including water, soil and wild plants (dandelions), and muscle tissue and offal of wildlife. Concentrations of heavy metals Cd, Pb, Hg and metalloid As were measured in the kidneys, liver and muscles of wild boars in three areas of the Papuk Nature park. Also, the paper investigated some of the possible sources of metal contamination in the Papuk Nature Park, including water, soil and wild plants (dandelion). Although essential heavy metals have some common properties in metabolic behaviour with the nonessential ones, essential elements have very important functions in living organisms, which lead to fundamental differences in approach and the interpretations of the results. Most of the research on wild boar meat samples refers to the concentrations of Cd and Pb, and much less to the concentrations of As and Hg. However, results of analysis and literature values for chromium, cooper, iron, zinc and vanadium concentrations in wild boar muscles, liver and kidneys are very scarce. Therefore, their concentrations will also be presented in this paper, and the obtained results will be compared with available literature data.

## 2. Materials and Methods

### 2.1. Papuk Nature Park

It covers an area of 336 km^2^ (33,600 ha) and is administratively located on the territory of two counties, Požega-Slavonska and Virovitica-Podravina.

### 2.2. Collection of Samples

Wild animal samples included muscle tissue and edible offal (kidney and liver) of wild boars. The research covered the entire area of the Papuk Nature Park. A total of 38 individuals were analysed: 17 muscle, kidney and liver samples were collected from the western part of the Nature Park, 10 samples each from the central and northern parts, and 11 samples from the southern part. The maximum age of the animals was 3 years.

For this experiment, wild boar meat was provided by the Voćin Hunting Society, which holds an annual hunting permit from the Government to reduce the number of wild predators in nature, such as wild boars. This meat was intended for distribution among members of society or for sale. This research is part of a study that is part of an ongoing project investigating the long-term impacts of war on the population and environment in Croatia and has been approved by the Ethical committee at Faculty of Medicine Osijek (project nr. 219-1080315-0288).

In addition, samples of soil (N = 31), water rivers, streams (N = 21) and wild plants (dandelion, N = 31) were sampled from the peripheral parts on the northern, southern, western and eastern sides of the Nature Park, as well as from its central part (total of 87 samples).

### 2.3. Sample Preparation

The meat samples were homogenised, weighed (0.5 g) and digested with concentrated HNO_3_ (Trace Metal Grade, Carl Roth, Karlsruhe, Germany) and 30% H_2_O_2_ (Trace Metal Grade, Carl Roth, Karlsruhe, Germany) using a microwave oven (Milestone UltraWAVE ECR). Ultrapure deionised water was used (Arium, purification system). The samples were heated for 40 min to a temperature of 200 °C and then held at that temperature for 30 min. After digestion, the vessels were cooled to room temperature. The samples were transferred to a pre-cleaned 25 mL volumetric flask, ultrapure water was added to volume and mixed with a vortex mixer before ICP-MS analysis.

Soil samples were collected from predefined locations based on Gauss–Krüger coordinates. After removing the top layer of grass with a shovel, probes were used to extract soil from a depth of 5 to 50 cm. The samples were then placed into containers resistant to soil constituents. The collected soil was ground and sieved through a 2 mm sieve. Subsequently, 0.3 g of each sample was treated with 6 mL concentrated HCl and 2 mL of concentrated HNO_3_. The digestion vessels were then placed in a microwave for processing.

Water samples from watercourses intended for determination of heavy metals and metalloids were collected in clean 250 mL plastic bottles. Each bottle was first rinsed with the sample water before being preserve with 1.25 mL concentrated HNO_3_. After collecting, the samples were stored in a portable cooling box at a temperature of 6 ± 2 °C and transported to the laboratory for analysis. In the laboratory, the samples were filtered using a membrane filter with a pore size of 0.45 µm. A 25 mL water sample was acidified with 2.0 mL of concentrated HNO_3_ and 6.0 mL of concentrated HCl (Trace Metal Grade, Carl Roth, Germany), cooled at room temperature and ultrapure water was added to volume.

Dandelion (*Taraxacum officinale*) leaves were been collected and packed into containers for further analysis. Leaves were rinsed with ultrapure water. Prior to digestion 1.0 g of sample was diluted with concentrated HNO_3_ and concentrated H_2_O_2_ (3:1), digested using HNO_3_ and HCl (5:1) and, after digestion, heated at 200 °C for 15 min. After heating, the samples were diluted to final volume of 10 mL with ultrapure water.

### 2.4. ICP-MS

After all the intended and planned samples were collected, they were transported by portable vehicle refrigerator, according to the recommendations of Dr. Andrija Štampar of the Health Ecology Service of the Teaching Institute for Public Health in Zagreb. They were first prepared for analysis, and then analysed in the laboratory for the mentioned heavy metals using the ICP-MS analysis method. ICP analyses were performed using a standard procedure according to accredited methods.

The settings of the ICP-MS were as follows: voltage power (RF), 1050 W; argon > 99.99% (Messer, Sulzbach, Germany). The phases and speeds of flow through the torch were as follows: (1) for the phase of plasma flow between the external and middle columns, speed 15.00 L/min; (2) for the phase of auxiliary flow of gas, speed 1.20 L min^−1^; (3) for the phase of argon gas nebulizer flow through the induction column, speed 0.88 L min^−1^.

All samples were analysed by mass spectrometry, with inductive coupled plasma (ICP-MS ELAN DRC-e, Perkin Elmer, Waltham, MA, USA). The instrument was recalibrated after every 12th sample, using an external standard (71-Element Group Multi Element Standard Solution, Inorganic Ventures, Christiansburg, VA, USA), with the use of internal standards with elements Y, In, Tb and Bi (Inorganic Ventures, USA). Internal calibration (an international laboratory audit) was conducted in cooperation with IFA Tulin (Department of the University of Natural Resources and Applied Life Sciences, Vienna, in cooperation with the Vienna University of Technology and the University of Veterinary Medicine). Analytical methods were validated with standard reference materials and standard samples. Polyatomic interferences with elements Fe, As and Cr were removed in the dynamic reaction chamber of the instrument, with reactive methane gas (CH_4_).

All steps of sample preparation and analysis were completed in a laboratory with standard heating, ventilation and air conditioning (HVAC) systems combined with high-efficiency particulate air (HEPA) filters. Commercially available reference materials used are as follows: Reference material ICP multielement solution for surface water testing X (Merck KGaA, Darmstadt, Germany), certified reference material RTC80 (Sigma Aldrich, St. Louis, MO, USA), certified reference material 1573a Tomato Leaves (NIST) and certified reference material ERM-CE278k (Sigma-Aldrich, St. Louis, USA) were used for checking the analytical accuracy of the measurement.

### 2.5. Statistical Methods

The median, maximum, minimum and confidence interval (CI) were used for the description of the data. One of multivariate data processing method, principal component analysis (PCA), was also used. The results were processed in Statistica (Tibco), version 14.0.0.15.

## 3. Results

Heavy metals such as Pb, Cd and Hg enter the environment from anthropogenic and natural sources which, due to their widespread presence in the environment, accumulate and act toxically in organisms of various species. They are characterised by a long biological half-life, entry into the food chain, and accumulation in biological organisms, and thus affect ecosystems with a certain toxic impact on the organism. Measuring the concentration of heavy metals and metalloids in the tissues of wild animals provides insight into environmental pollution, possible immunopathological changes, and intoxication of wild animals. It also gives insight into the healthiness of food for human consumption. Wild boar meat, intended for human consumption, must meet certain quality conditions prescribed by the Ordinance on the Maximum Permitted Amounts of Certain Polluting Substances in Food.

The results are presented in the following tables (Table 1, Table 2 and Table 3), which show the median values, minimum–maximum values and 95% confidence intervals (CIs), as well as the recommended values and the percentage of samples that exceeded the recommended values of the analysed elements in different tissues of wild boars.

**Figure 2:**
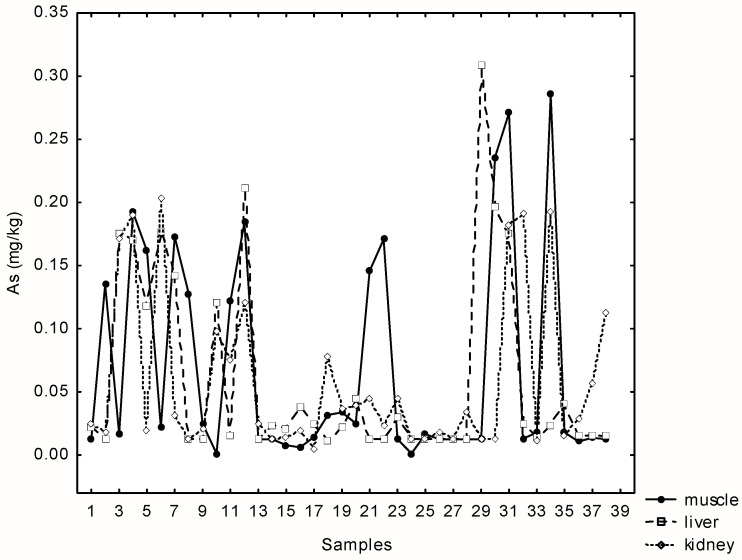
Relationships between arsenic concentrations in liver, kidney and muscle tissue samples of 38 wild boars at Papuk Nature park, Croatia.

The concentration of arsenic was below the detection limit (0.0125 mg kg^−1^) in 21% of the total samples. Very similar maximum concentrations of arsenic were recorded in the liver, kidney and muscle samples (range 0.214–0.303 mg kg^−1^). Statistically significant and positive correlations were found between As in liver, kidney and muscle samples (r**_s_**_(muscle-kidney)_ = 0.33; r_**s**(muscle-liver)_ = 0.34, *p* = 0.00; r_**s**(kidney-liver)_ = 0.4; *p* = 0.00) (Figure 2).

There is no maximum permissible level of As in meat prescribed in the EU, so we compared the values with those published by the Croatian regulatory agency from 2003 (see MAC values in Table 1, Table 2 and Table 3). Unlike muscles and liver, where both, median and maximum concentrations are below those allowed, concentrations in muscles exceeded the maximum permitted limit (0.1 mg kg^−1^) in 30% of cases.

Cadmium concentrations were above the detection limit (0.0025 mg kg^−1^) in all tissue samples. Maximum Cd concentration was 0.672 mg kg^−1^ in the muscle, 4.60 mg kg^−1^ in the liver and 6.64 mg kg^−1^ in the kidneys of wild boars.

As in the case of arsenic, there is no maximum permissible European Union-prescribed concentrations of Cd in game meat, although in livestock meat products, the maximum levels for cadmium are 0.05 mg kg^−1^ (muscle), 1 mg kg^−1^ (kidney) and 0.5 mg kg^−1^ (liver).

**Figure 3:**
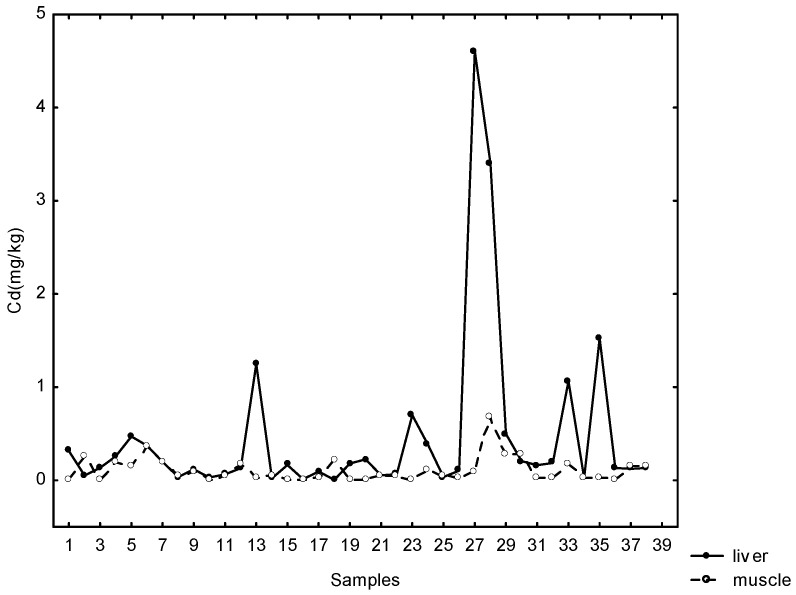
Relationship between cadmium concentration in liver and muscles.

Fairly large percentages—42% of muscle, 44% of kidney and 18% of liver samples—contained Cd concentrations above the permitted values. Weak positive statistically significant correlations were found between Cd in liver and muscle samples (r_**s**_ = 0.35; *p* < 0.05) (Figure 3).

Based on the proposal of the Commission Regulation (EU, 2018/73), the maximum level of Hg in meat should not be higher than: 0.01 mg kg^−1^ (muscle), 0.02 mg kg^−1^ (kidney) and 0.02 mg kg^−1^. The results showed that the most affected organs are liver (max. = 0.552 mg kg^−1^) and kidney (max. = 0.484 mg kg^−1^) and the least affected are muscles (max. = 0.103 mg kg^−1^) [22]. In this study, 15%, 27% and 13% of the kidney, liver and muscle samples contained higher Hg levels than the maximum level set for domestic pig meat and offal in the EU Commission Regulation.

Pb was detected in all samples. The highest Pb concentrations were measured in the kidney (11.8 mg kg^−1^), then the muscles (9.10 mg kg^−1^) and lastly the liver (0.463 mg kg^−1^). The maximum concentrations in muscles (65%), kidney (51%) and liver (63%) exceeded the limits prescribed by EU regulations [22].

The maximum lead concentration in this study was 11.8 mg kg^−1^ (kidney) and 9.10 mg kg^−1^ (muscle), but this was the case in two different individuals. In other samples, lead concentrations in kidney and muscles ranged from 0.040 mg kg^−1^ to 1.80 mg kg^−1^, and 0.030–1.65 mg kg^−1^ respectively. We did not find any statistically significant correlations between the concentrations of Pb in muscles, kidney and liver. Environmental concentrations of metals in soil, water and dandelion are shown in Table 4.

In order to detect environmental contamination, dandelion could be used as a proper material for laboratory testing [18,24]. Dandelion is an excellent bio-indicator for heavy metal contamination in environments, and in this study was chosen because it was common to all of the sampling sites. While the mean and maximum concentrations of As in water were within the permissible values (10 µg L^−1^), the maximum concentrations of arsenic in dandelions on the southern side of Papuk were slightly elevated compared to the old regulation on As concentrations in leafy vegetables (MAC—maximum allowed concentration according to Croatian legislation = 300 µg kg^−1^).

In samples taken from central part of Papuk area, maximum arsenic values (36.0 mg kg^−1^) that were a few times above European topsoil content of 12.0 mg kg^−1^ (Table 4) were recorded. Higher concentrations of Cd in dandelion samples are associated with the northern side (max. = 1122 µg kg^−1,^ MAC = 100 µg kg^−1^), while higher concentrations of Pb (max. = 2583 µg kg^−1^, MAC = 300 µg kg^−1^) are associated with the southern side of the Papuk Nature Park.

The median and maximum Cd, As, Pb and Hg concentrations in rivers and tributaries were below Croatian maximum admissible levels of 5 µg L^−1^, 10 µg L^−1^, 5 µg L^−1^ and 1 µg L^−1^.

The results presented in Table 5 show the median, minimum and maximum values of Cr, Cu, Fe, V and Zn concentrations in muscles, kidneys and livers of wild boar.

Comparing the concentrations of Cr, Cu, Fe and Zn in water and soil with reference ranges and concentrations in dandelions analysed in other national parks, we can see that there are no major deviations in comparison to areas that were not exposed to anthropogenic influences [24,25,26].

In order to investigate the existence of correlation between samples and variables (metals), as well as possible differences between samples with regard to the parts from which they were collected, we applied the principal component method (PCA) to the observed data.

Due to the complexity of the numerous relationships in the data matrix, it was difficult to draw clear conclusions directly. Principal component analysis methods can extract the hidden information and explain the structure of the complex data sets. The results of PCA revealed two significant principal components that explained ca 80% of the total variance of the data set.

**Figure 4:**
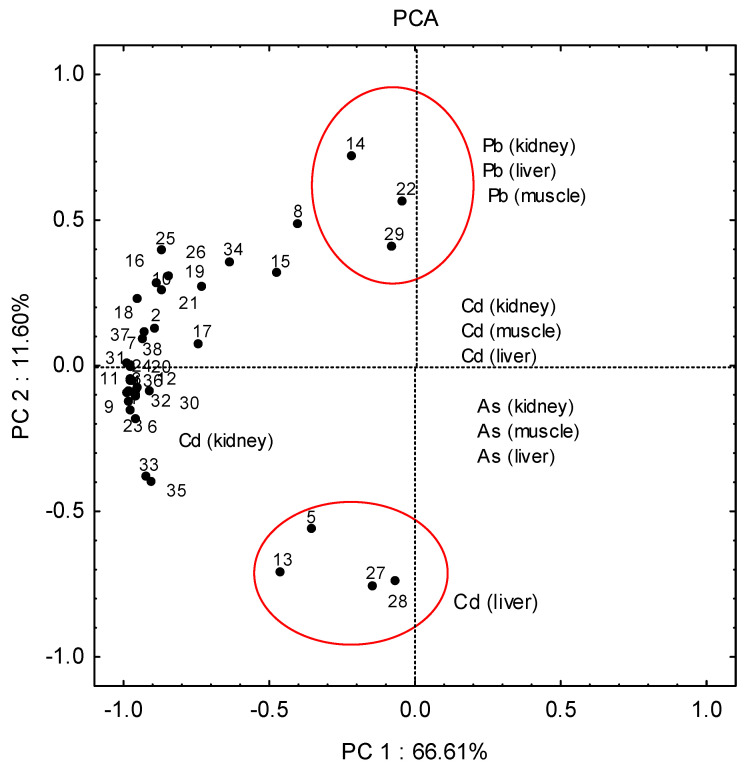
The results of principal component analysis (PCA) of 114 samples of liver, kidney and muscle from three areas of Papuk (west, south, and central–northern areas).

Principal component analysis of 114 samples from three areas of Papuk (west, south, and central–northern part) showed a very weak separation of samples considering the different areas from which they were sampled (Figure 4). However, three main mixed clusters of samples can be distinguished: A rather compact cluster in the left-hand portion of Figure 4 contains the majority of samples. Since the variables (metals) that point toward certain objects are more important for those objects, it is obvious that they are characterised by higher concentrations of cadmium in the kidneys.

A few samples were separated into a second separate mixed cluster (13-west, 5-west, 27-south, 28-south), and were characterised by higher concentrations of cadmium in the liver. The second, poorly defined cluster also composed of samples from different parts of the research area (14—west, 22—central–northern, 29—south), was characterised by higher concentrations of lead in the liver, kidneys and muscles. The remaining samples are in a complex cluster characterised by higher concentrations of cadmium in the kidney.

## 4. Discussion

Game meat belongs to those foods that are less often consumed by the majority of the general population but are more often consumed by certain subgroups of the population (hunters and their family members, relatives and friends). This meat is becoming increasingly available to consumers through restaurants, supermarkets, and high street butchers, and is often promoted as a healthy, “wild” and “organic food” alternative to commercially produced pork [27].

The presence of metals like Pb, Cd, Hg and As in higher concentrations could be dangerous to the environment and, subsequently, to animal meat and, later, to consumers. In contrast to other food items, game meat has an additional factor influencing heavy metal content due to the use of hunting ammunition [28]. In general, the results of our research showed that the metal that exceeded permitted limits in the largest number in liver, kidney and muscle samples was Pb, followed by Cd, followed by Hg and finally As (only in muscles). Although the median values (with exception of that of Pb) were, in most cases, below the limits of the permissible values, the maximum concentrations of most of the analysed elements were from several times to several tens of times higher than the permissible ones. Therefore, concentrations of these metals above concentrations suggests that there is a good reason to investigate possible sources of contamination.

Maximum As concentration was 0.286 mg kg^−1^ in the muscle, 0.306 mg kg^−1^, in the liver and 0.214 mg kg^−1^ in the kidney of wild boar. Even 30% of muscle samples contained maximum As concentrations above the permitted values (MAC = 0.1 mg kg^−1^).

Similarly to our results, maximum arsenic concentrations were observed in wild boar muscle (0.31 mg kg^−1^), kidney (0.82 mg kg^−1^) and liver (0.9 mg kg^−1^) samples in meat in Slovakia and Hungary (<0.5 mg kg^−1^) [29,30]. Lower concentrations of As in kidney were found in Swedish studies (<0.08 mg kg^−1^) but much greater concentrations (0.06 to 2.94 mg kg^−1^) were found in a relatively clean area—Tatra National Park—which is legally protected and restricted with regard to all industrial activities [27,31]. In comparison with our results, the results of previous study conducted in three counties of eastern Croatia showed ten times lower maximum concentrations of arsenic in muscle (0.067mg kg^−1^), kidney (0.054 mg kg^−1^) and liver (0.068 mg kg^−1^) [32]. The occurrence of arsenic in elevated concentrations in the local water supply systems of eastern Croatia is not an isolated phenomenon, because it is a systemic hydrogeochemical phenomenon that is characteristic of the wider area of the Danube and Drava Rivers [33].

The spatial distributions of the total groundwater arsenic concentrations are not homogenous over the area of eastern Croatia. Higher concentrations in groundwater and plants were found in Osijek and surroundings than in Baranja and western part of Osijek, Baranja County [34]. Higher concentrations of arsenic in the area of Papuk were also recorded in previous geological surveys [16]. The analysis of major, minor and trace elements in mineral samples from Radlovac creek (eastern part of Papuk area) showed two thousand times higher arsenic concentrations when compared to normal values that could be obtained in sandstone sediments. In the Pannonian region of Croatia, the content of total arsenic varies from 0.9 mg kg^−1^ soil up to 490 mg kg^−1^ soil in the surface layer of agricultural soil [35]. Since the highest measured As concentrations in muscle, kidney and liver were determined in the same individuals (northern part), acute intoxication can be eliminated as a source, and only chronic exposure with additional metal burdening can be discussed.

Hg concentrations in wild boar tissues should be low, which is characteristic of terrestrial animals. However, in contrast to the median values, which were all within the permissible values, the maximum values in this study were above the permissible concentration in muscle, kidney and liver samples. Mercury was detected in 93% of all samples, which demonstrates that this element is still present in the environment and can accumulate in animal tissues. The most attacked organs are kidney (excess 27%) and muscles (excess 15%), and the least affected is liver (excess 13%). With minor exceptions, the maximum concentrations of Hg in tissues of wild boars were similar to values previously reported in Hungary, Slovakia, Croatia and Serbia [30,31,32,36,37,38]. However, some differences have been observed. For example, despite similar maximum mercury values in the kidneys 0.484 mg kg^−1^ (Papuk) and 0.56 mg kg^−1^ (Serbia, Morović), the maximum recorded mercury values in liver in the Papuk area (0.552 mg kg^−1^) were several times higher than those described in Serbia (0.08 mg kg^−1^) [38].

Although our maximum values for Hg concentrations in the liver and muscles are similar to the concentrations from three counties in eastern Croatia, kidney concentrations in the Papuk area are a few times lower (max = 0.484 mg kg^−1^). The data published by Polish authors with regard to Hg concentrations in organs (max. liver 0.06 mg kg^−1^) of wild boars from unpolluted areas were remarkably lower than the data from the present study (Papuk 0.552 mg kg^−1^) [39]. By analysing the mercury concentrations in soil, rivers, streams and dandelions taken from area of the Papuk Nature Park, it was determined that the values correspond to the values found in areas that did not have elevated Hg concentrations in the environment [25]. Looking at soil, water (from rivers and streams) and plant samples together, it can be observed that all median concentrations were within permitted values.

Higher concentrations of Hg in the liver (0.48 mg kg^−1^) and kidney (1.6 mg kg^−1^) of wild boars from the contaminated area of central Zemplin in the Slovak Republic attributed to anthropogenic influences, mainly industry, agriculture and traffic [29]. Considering that there is no industry, agricultural activities and traffic in the area of the Papuk Nature Park, nor is the area populated, according to current knowledge, we cannot attribute higher concentrations of mercury in liver, kidney and muscles to any human activities.

In deeper layers of soil, mercury most often binds to clay minerals, especially montmorillonite and kaolinite. In upper layers, mercury and its complexes bind to organic matter. In this part of Croatia, mercury concentrations in soil range between 0.005 and 0.850 mg kg^−1^, with a median of 0.040 mg kg^−1^. Increased concentrations of this element have also been registered in soils lying on the alluvium deposits of the Sava River and in higher parts of Papuk and Krndija mountains. North of the town Đakovo (at a distance of 80 km from Papuk) an isolated locality with higher Hg concentrations in soil was observed. At some locations, Hg contamination was the consequence of anthropogenic sources, for example, in the middle section of the Krka River estuary. A hot spot was found in the soil at one location in the town of Sisak [40]. Other parts of the region have mercury concentrations in soils below the median value [26]. Given that the higher concentrations of Hg in the area of eastern Croatia are of epigenetic origin, higher concentrations of mercury in the tissues of wild boars cannot be associated with anthropogenic influences.

Cadmium and lead concentrations in all investigated tissues were above limits of quantification (0.0025 mg kg^−1^ and 0.0022 mg kg^−1^). Maximum Cd concentrations in liver (4.60 mg kg^−1^), kidney (6.64 mg kg^−1^) and muscle (0.672 mg kg^−1^) were similar to those reported in previous study conducted in the same County during period 2006–2007 [37]. For comparison, data published by Poland with regard to toxic element presence in organs of wild boars from unpolluted areas show significantly lower Cd concentrations, from 0.02 mg kg^−1^ to 0.06 mg kg^−1^ (liver) and 0.07 mg kg^−1^ to 0.15 mg kg^−1^ (kidney) [39]. Median and maximum concentrations of Pb and Cd in water and soil were within the permitted values, while the concentrations of Pb and Cd in dandelion on the southern and central part of Papuk Nature Park were elevated (max. Pb = 2583 µg kg^−1^, max. Cd = 1122 µg kg^−1^).

Cadmium is accumulated predominantly in the kidney and ovary, bound to the protein metallothionein and it is suspected to be an endocrine disruptor, mimicking oestrogen. In vitro studies on wild boar further showed that Cd^2+^ exposure impairs immune cell function, which may increase susceptibility to infection [27,41]. Cadmium is carcinogenic, adversely affects the kidney, bones, cardiovascular and immune system and belongs to Group I according to the IARC classification [42]. The wild boar is an omnivore and about 90% of their diet consists of plant matter, but animal matter and fungi are also found [43]. Plants react differently to different concentrations of cadmium. Possible abnormalities due to its harmful impacts depend on the period of time that the plant was exposed, the amount of cadmium intake, its localization in plant parts and the type of plant itself. Cadmium can localise anywhere in plants due to phloem transport. It is known that mushrooms accumulate certain metals, for example, Cd^2+^, Cd^6+^, Hg^2+^ and As^5+^ in their fruiting bodies. Concentrations in uncontaminated areas are in the range from <0.5 mg kg^−1^ to 2 mg kg^−1^, while concentrations in contaminated areas were as high as 10 mg kg^−1^ [44].

The analysis of Cd, Hg, Pb and As in other plants, including mushrooms, in the area of the Papuk Nature Park should definitely be the next step in the continuation of research into the exposure of wild animals to metals. Since the highest measured Cd concentrations in muscle and liver (*r*_s_ = 0.34) were determined in the same individual, this indicates systematic contamination. Wild boar rooting directly alters soil structure and the most obvious direct effect of rooting by wild boar is reduction in plant cover [43]. Roots are an efficient barrier for metals passing into the upper parts of plants; thus, it can be assumed that wild boars that dig up plants with their snouts and eat food derived from these plants will accumulate more metals and metalloids, such as arsenic [14].

According to Scheuhammer (1987), it is very preferable to measure cadmium concentrations in the liver and kidneys because the ratio of cadmium concentrations between these two tissues can provide additional information [45]. If the liver/kidney concentration ratio is >1, this result indicates acute exposure to relatively high doses of Cd, while liver/kidney ratios < 1 indicate a situation of chronic low-level exposure. The relationship between cadmium concentrations in liver and cadmium concentrations in kidney of the 38 sampled wild boar showed that the liver/kidney ratio was <1 in 88% of the analysed subjects. Similarly to the results of Franzoni et al., our results support the thesis of chronic exposure to cadmium, since the liver is the first site of cadmium absorption [41]. Higher concentrations of Pb in tissues were found only in two samples. Other values were significantly lower and ranged around the median value. Furthermore, unlike arsenic, high concentrations of Pb in tissues were not found in the same individuals, therefore the possibility of systematic contamination is excluded. Pb bullets are now known to fragment as they pass into/through game, and hundreds of tiny fragments may radiate into tissues up to distances of 45 cm from the bullet path [46]. Although our muscle lead concentration results were not similar to those reported in Europe, where Pb was very high (range 324.6–1095.9 mg kg^−1^) around the bullet entry zone, it is possible to assume that the higher lead concentrations are the result of ammunition that has a tendency to fragment and scatter through the hunted organism [12].

By analysing the arsenic, cadmium, mercury and lead concentrations in soil, water and dandelion taken from the area of the Papuk Nature Park, it was determined that the values correspond to values of concentrations found in areas that did not have elevated metal and metalloid concentrations in the environment [25]. Surprisingly, the maximum concentrations of cadmium, lead and arsenic in the soil samples are several times higher in the Papuk Nature Park than in the industrial and agricultural areas of eastern Croatia [19]. It is known that wild pigs travel long distances and are not confined to a strictly defined area, which makes it impossible to find correlations between metal concentrations in tissues and those in the environment. However, it was expected that the analysis of water, soil and dandelions would provide additional information about possible “accumulations” of certain elements in different parts of the world of the Papuk Nature Park, which could possibly explain their concentrations in the tissues of hunted animals. Except for the correlation of higher cadmium concentrations in dandelion and soil, no other regularities were observed. Higher concentrations of arsenic in organs were observed on the western side, while higher concentrations of arsenic in dandelions were found on the southern side of the research area. At the same time, higher concentrations of arsenic in the soil were recorded in the central part, and in water in the northern part. The absence of correlations between metal concentrations in water, soil, organs and dandelions, with respect to the sides of the study area, is also present in the case of Cd, Hg and Pb. The result of PCA did not indicate the occurrence of grouping of samples with regard to the three areas of Papuk from which the samples were collected (the west, south, and central parts).

Higher concentrations in the Papuk area environment cannot be connected with anthropogenic influences such as crossroads, industry, herbicide, pesticides, etc., because there were no permanent residents in the area, there was no agricultural activity, and there was no industry or heavy traffic. Lead, cadmium, mercury and arsenic are present in the ecosystem of eastern Croatia. Wild boars are an omnivorous species and free-migrating animals; thus, they can move long distances through the day, taking in the environmental contamination of large areas.

Although essential heavy metals have some common properties in metabolic behaviour with the nonessential ones, essential elements have very important functions in living organisms, which leads to fundamental differences in the approach and interpretation of the results.

The values of 0.14 mg kg^−1^ provided in Italy and 0.13 and 0.14 mg kg^−1^ in Hungary for chromium concentrations are in good agreement with the 0.193 mg kg^−1^ (muscle), 0.140 mg kg^−1^ (kidney), 0.130 mg kg^−1^ (liver) found in our study [30,47].

The detected Zn concentrations were in a range from 12.1 to 47.44. mg kg^−1^ in the muscles from Slovakia, Hungary and Germany, which shows similar values to our study—i.e., 20.6 mg kg^−1^ (muscle), 23.3 mg kg^−1^ (kidney) and 36.0 mg kg^−1^ (liver) [30,48,49].

Besides zinc, iron has the highest median concentration of trace elements, with a value of 44.5 mg kg^−1^ (muscle) which is in close agreement with the scarce data in the literature. Dannenberger et al. (2013), Falandyzs et al. (1994) and Ertl et al. (2016) reported similar Fe concentrations from 19 to 54 mg kg^−1^ [50,51,52]. The concentrations of copper (2.94, 5.32 and 3.71 mg kg^−1^) are also in agreement with previous studies conducted in Slovakia (1.62 mg kg^−1^), Hungary (1.22 mg kg^−1^) and Germany (1.31 and 1.36 mg kg^−1^) [30,48,49].

Chromium, copper, iron and zinc concentrations, found in our investigations, were similar to those reported in the studies made on the wild boar population in other European countries. In comparison with results of investigations in another part of EU, our results are promising since they suggest that the ecosystem in eastern Croatia is stable concerning Cr, Cu, Fe and Zn pollution. The results of this work indicate that the entrails and muscles of such relatively young animals (up to 3 years of age), as investigated in this work, are not suitable for consumption due to the high concentrations of Cd, Pb, Hg and As in the muscles. When all measured values are taken into consideration, meat from these animals should be consumed with caution; however, measured values do not represent a risk of potential acute intoxication from medical point of view. Further extended analysis should be carried out, and strict surveillance of game animals should be maintained. Unfortunately, despite the fact that wild boar meat is considered a delicacy and is consumed in larger proportion, there is no systematic analysis of metals. Contamination in this case could not be attributed to war nor war associated elements; however, it could be considered as an additional burden in this case. Further research should focus on analysis of the meat and entrails of older animals, as well as analysis of the food the animals are fed during the winter period (corn). The research should certainly also include other plant species that wild boars consume (oak nut, roots, mushroom, etc.).

## 5. Conclusions

This study was the first to examine the exposure of wild boars from the Papuk area to heavy metals (Cd, Hg and Pb) and the metalloid As. A fairly large concentration of toxic elements in the kidneys, liver and muscles of these animals exceeded the levels considered acceptable for human consumption. The results of analysis showed a prevalence of contaminated meat and offal samples in all investigated locations of the Papuk Nature Park (west, south and central–northern). Since the area of the Papuk Nature Park is uninhabited and there is no industry or roads, elevated concentrations of metals in the tissues of wild boars could not be related to anthropological influences. Metal concentrations are difficult to compare due to different measurement methods, different ages of animals and different values with which they are presented (median, mean, range, percentile). Comparison of the results obtained from this research with reports from other parts of Croatia showed that the median values are of similar orders of magnitude. However, larger differences in concentrations were manifested mostly in the ranges of maximum values. In addition to the commonly known elevated concentrations of As, Cd, Hg and Pb in the kidneys and liver, the levels of arsenic, cadmium and mercury in the muscles are of greatest concern. Further research is needed over a longer period of time to achieve a clearer picture of the real situation. In any case, measures applied in the natural park regime will certainly contribute to a lower burden on the environment.

## Figures and Tables

**Table 1:** Arsenic, cadmium, lead and mercury content in muscles (mg kg^−1^ wet weight).

Muscle (N = 38)						
Variable	MAC	Excess %	Median	Minimum	Maximum	CI
As	0.1 **	30	0.017	<0.012	0.286	0.040–0.090
Cd	0.05 *	43	0.047	0.004	0.672	0.060–0.151
Pb	0.1 *	65	0.15	0.03	9.1	0.006–0.962
Hg	0.01 ***	15	0.014	<0.005	0.103	0.010–0.020

* MAC for cadmium and lead concentrations [22]; ** MAC for arsenic concentrations [17]; *** MAC for mercury concentrations [23].

**Table 2:** Arsenic, cadmium, lead and mercury content in kidney (mg kg^−1^ wet weight).

Kidney (N = 38)						
Variable	MAC	Excess %	Median	Minimum	Maximum	CI
As	0.5	0	0.028	<0.0125	0.214	0.037–0.079
Cd	1	50	0.972	0.057	6.64	1.15–2.24
Pb	0.15	51	0.169	0.042	11.8	0.054–1.19
Hg	0.02	27	0.076	<0.005	0.484	0.060–0.121

**Table 3:** Arsenic, cadmium, lead and mercury content in livers (mg kg^−1^ wet weight).

Liver (N = 38)						
Variable	MAC	Excess %	Median	Minimum	Maximum	CI
As	0.5	0	0.02	<0.0125	0.306	0.030–0.080
Cd	0.5	20	0.145	0.005	4.6	0.152–0.765
Pb	0.15	63	0.196	0.013	0.463	0.162–0.233
Hg	0.02	13	0.02	<0.005	0.552	0.020–0.092

**Table 4:** The concentrations of selected elements in soil, water and dandelion samples.

Element	Water (µg L^−1^) Median (max.)	Soil (mg kg^−1^) Median (max.)	Dandelion (µg kg^−1^) Median (max.)
Cd	0.211 (0.523)	0.245 (12.0)	271 (1122)
Pb	2.64 (4.82)	11.1 (42.0)	492 (2583)
As	1.51 (3.03)	6.22 (36.0)	77.8 (495)
Hg	0.094 (0.952)	0.100 (0.100)	0.030 (0.100)
Cr	2.90 (10.5)	2.94 (27.3)	699 (17,333)
Cu	1.56 (16.1)	5.44 (31.5)	10,521 (17,373)
Fe	190 (2 422)	17,774 (31,404)	145,882 (563,190)
Zn	17.2 (225)	57.3 (124)	48,144 (72,012)

**Table 5:** Descriptive statistics of the elements analysed in muscles, kidneys and livers of wild boars (mg kg^−1^ wet weight).

Element (mg kg^−1^)	Cr Median (min.–max.) CI	Cu Median (min.–max.) CI	Fe Median (min.–max.) CI	Zn Median (min.–max.) CI
Muscle	0.193	2.94	44.5	20.6
	(0.020–1.04)	(1.24–6.32)	(7.87–187)	(6.30–61.3)
	(0.164–0.295)	(2.70–3.38)	(36.7–60.1)	(19.3–27.2)
Kidney	0.140	5.32	110	23.3
	(0.010–1.55)	(2.20–14.7)	(25.2–612)	(4.20–88.5)
	(0.140–0.310)	(5.09–6.09)	(92.3–182)	(20.3–29.1)
Liver	0.130	3.71	278	36.0
	(0.020–2.70)	(1.20–19.8)	(21.3–921)	(4.63–70.4)
	(0.080–0.461)	(3.33–5.87)	(241–416)	(29.9–40.8)

## Data Availability

The data supporting this study’s findings are available from the cor-responding author upon reasonable request.
